# The Safety of Propofol Versus Sevoflurane for General Anesthesia in Children: A Meta-Analysis of Randomized Controlled Trials

**DOI:** 10.3389/fsurg.2022.924647

**Published:** 2022-06-22

**Authors:** Ying Zhao, Feng Qin, Yuhang Liu, Yanping Dai, Xiaobo Cen

**Affiliations:** ^1^ National Chengdu Center for Safety Evaluation of Drugs, State Key Laboratory of Biotherapy/Collaborative Innovation Center for Biotherapy, West China Hospital, Sichuan University, Chengdu, China; ^2^ Andrology Laboratory, Department of Urology, West China Hospital, Sichuan University, Chengdu, China

**Keywords:** general anesthesia, children, meta-analysis, propofol, sevoflurane

## Abstract

**Background:**

Propofol and sevoflurane are the most used anesthetics for pediatric surgery. Emergence agitation, postoperative nausea and vomiting and postoperative pain are the primary adverse effect of these general anesthetics. Many clinical studies had compared the safety of propofol and sevoflurane in pediatric surgery, but the results were controversial.

**Objectives:**

To evaluate the evidence surrounding the safety of propofol versus sevoflurane for general anesthesia in children.

**Methods:**

Databases including PubMed, Embase, Cochrane Library, China National Knowledge Infrastructure, Wanfang Data and Vip Data were searched to collect relevant articles. Trials were strictly selected according to previously defined inclusion and exclusion criteria. RevMan 5.3 software was used for meta-analyses.

**Results:**

Twenty randomized controlled trials recruiting 1,550 children for general anesthesia were included, with overall low-moderate methodological quality. There was evidence that compared with sevoflurane anesthesia, propofol anesthesia significantly decreased the incidence of emergence agitation (OR = 4.99, 95% CI, 3.67–6.80; *P* < 0.00001), postoperative nausea and vomiting (OR = 1.91, 95% CI, 1.27–2.87; *P*  =  0.002) and postoperative pain (OR = 1.72, 95% CI, 1.11–2.64; *P* = 0.01) in children. However, patients who received sevoflurane tended to have shorter times to eye opening (MD = −2.58, 95% CI, −2.97– −2.19; *P* < 0.00001) and times to extubation (MD = −1.42, 95% CI, −1.81– −1.02; *P* < 0.00001).

**Conclusions:**

This review reveals that the children who received propofol anesthesia had the lower risks of emergence agitation, postoperative nausea and vomiting and postoperative pain when compared with sevoflurane anesthesia. But the children who received sevoflurane recovered slightly faster than those received propofol. Considering the limitations of the included studies, better methodological quality and large controlled trials are expected to further quantify the safety of propofol and sevoflurane for general anesthesia in children.

## Introduction

Each year, an estimated six millions of children, including 1.5 million infants, are exposed to general anesthesia during the course of surgery, imaging, and other medical procedures in the United States (1, 2). Childhood is characterized by numerous physiological changes and is the critical and sensitive period for brain development, which are easily affected by general anesthesia (3, 4). Compared with adult surgery, pediatric surgery often requires smaller trauma, shorter operation times and lower risk of postoperative complications. As a result, the safety of general anesthetics given to children is a critical public health concern.

Propofol and sevoflurane are the most used anesthetics in pediatric surgery (5–7). Propofol, a short-acting intravenous anesthetic, has the advantages of fast onset, rapid recovery, stability, and prevention of nausea and vomiting, which is widely used for the induction and maintenance of intravenous anaesthesia (6). Sevoflurane, a versatile inhalational anesthetic, has the advantages of rapid induction, easy control of anesthetic depth, quick recovery and limited respiratory stimulation, which is also widely used in paediatric anaesthesia (7). Despite the widespread use of these anesthetics, 26% of pediatric patients continue to experience emergence agitation (EA), 25% of pediatric patients continue to experience postoperative nausea and vomiting (PONV), 24% of pediatric patients continue to experience postoperative pain (POP), and some pediatric patients continue to experience the short-term memory impairment (8–11). At present, many clinical studies with small sample sizes had compared the risks of major complications in paediatric patients undergoing anesthesia with sevoflurane and propofol, but the results were controversial, and very little meta-analysis had been performed on this topic yet (7, 10–12).

Therefore, this study evaluated the available evidence of propofol and sevoflurane and took a meta-analysis by using the Cochrane system evaluation method. Specifically, the present study aimed to evaluate whether propofol was superior over sevoflurane in the incidences of EA, PONV and POP, and times to eye opening and extubation for general anesthesia in children. The information would be used to select the appropriate anesthetics for pediatric surgery in clinical practice.

## Materials and Methods

### Search Strategy

The study was registered in the PROSPERO database. A systematic search of PubMed, Embase, Cochrane Library, China National Knowledge Infrastructure (CNKI), Wanfang Data and Vip Data for studies on propofol and sevoflurane were performed. Dates ranged from the inception of the different databases through Mar 15, 2022. The search terms were “propofol OR diprivan OR propofolum” (Yi Bing Fen in Pinyin), and “sevoflurane OR sevo” (Qi Fu Wan in Pinyin). The search terms were applied in the following combinations: (sevoflurane OR sevo) AND (diprivan OR propofolum OR propofol) for English databases, and “Yi Bing Fen” AND “Qi Fu Wan” for Chinese databases. In this study, we investigated the safety of sevoflurane versus propofol in Children. The reference lists of existing articles as a supplementary method were further searched for relevant studies.

### Inclusion Criteria

Studies were included in this present meta-analysis if they met the following inclusion criteria: (1) Study design: all participants were randomly allocated to intervention groups, both parallel and crossover studies were included for eligibility eligible; (2) Population: all participants were children aged younger than 12 year, and require surgical intervention; (3) Comparison: studies had to compare propofol with sevoflurane, patients in each group can be given other sedative and analgesic drugs during the perioperative period; (4) Outcome: studies have used dichotomous data based on EA, or PONV or POP as outcome indexes.

### Exclusion Criteria

All case reports, animal studies, editorial comments, non-clinical outcome studies, and literature reviews were excluded. Case series or clinical trials regarding the safety of propofol and sevoflurane on children were also excluded if they: (1) were unverified randomized controlled trial (RCT); (2) did not meet all of the inclusion criteria; (3) had no original data available for retrieval; (4) were duplicate publications.

### Data Extraction

The articles were independently screened by two reviewers (YZ and FQ). From the included RCTs, data were extracted on the following outcomes when they were reported: title, the first author, publication year, country, study design, age of the participants, number of the participants, type of operation, American society of anesthesiologists (ASA) class, intervention measures (the name and dosage of the medication), and outcome indexes (such as EA, PONV, and POP). The data were verified by a third reviewer (YL). The information about the baseline was also extracted from the relevant articles. If necessary, the reviewers would try to obtain incomplete information from the study investigators.

### Bias Assessment

Two reviewers (YZ and FQ) independently evaluate the risk of bias by using the Cochrane Collaboration bias risk tool (13). The following factors were evaluated particularly: (1) random sequence generation; (2) allocation concealment; (3) blinding both participants and personnel; (4) blinding of outcome assessment; (5) addressing incomplete outcome data; (6) selective reporting bias; and (7) other biases.

### Selected Outcomes

Five predefined outcomes were assessed. The primary outcome was the incidence of EA after general anesthesia in children. The secondary outcomes were the incidence of PONV, the incidence of POP, the extubation time and the eye-opening time after general anesthesia in children.

### Statistical Analysis

Statistical analyses were performed with RevMan 5.3 software (Cochrane Collaboration, London, UK). The risk of bias of the included studies was further analyzed by the Cochrane Collaboration’s tool. The proper effect sizes and statistical analysis methods were chosen according to different data types and evaluation purposes. For continuous outcomes, the mean difference (MD) and 95% confidence interval (CI) were calculated. For discontinuous outcomes, odds ratio (OR) and 95% CI were calculated. We used fixed-effects models if there was no significant heterogeneity (I^2^ ≤ 50%, or *P* > 0.1). Otherwise, we used random-effects models. Publication bias was assessed by the funnel plot.

## Results

### Literature Search

**Figure 1:**
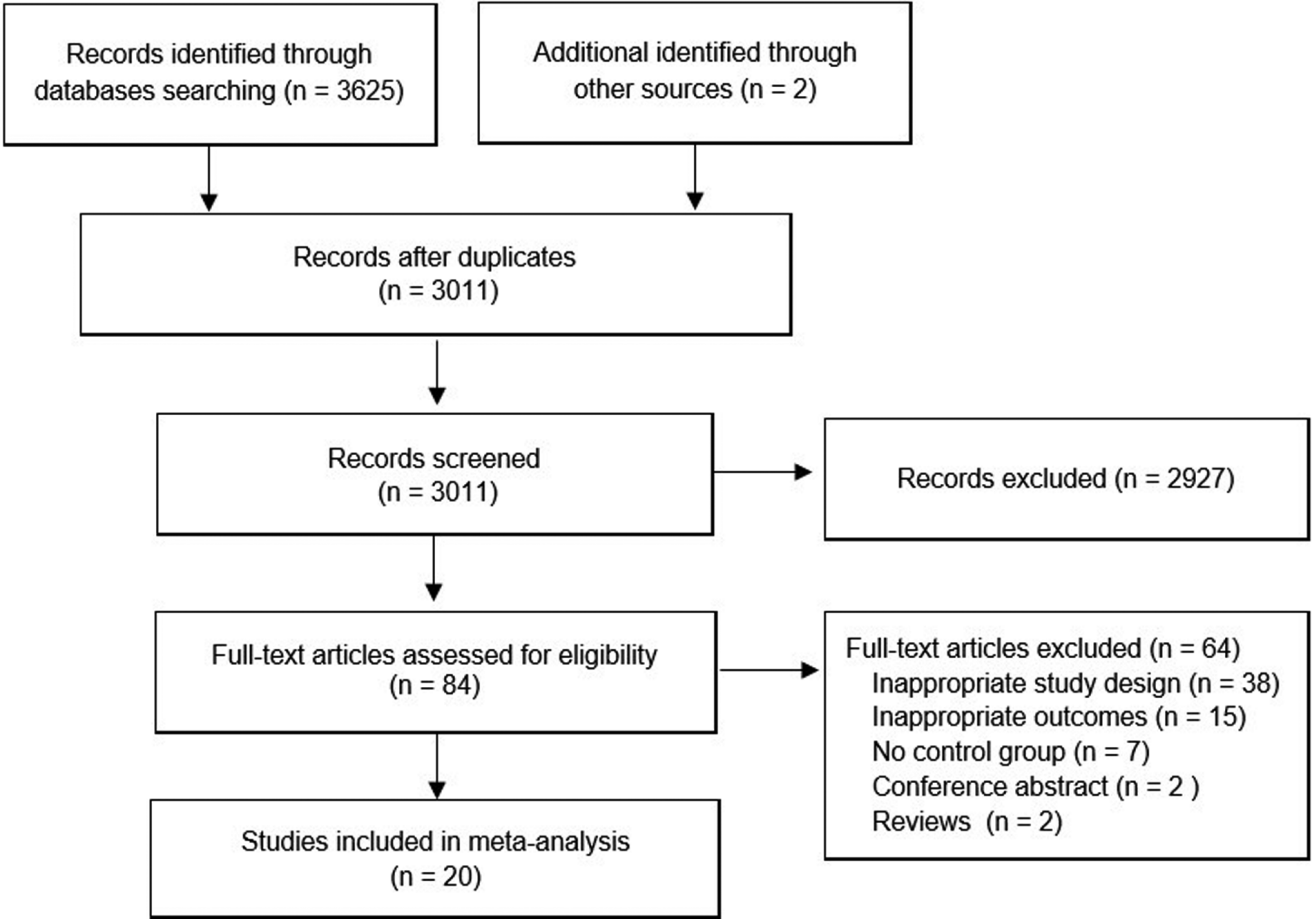
Study selection process for the meta-analysis with specifications of reasons.

An overview of the study selection process is presented in Figure 1. In total, 20 studies with 1,550 patients (783 for sevoflurane group, 767 for the propofol group) were finally included in the present study (10, 14–32). All studies were RCTs, and most of these RCTs were small (average sample size of 77.5); the studies were published between 1998 and 2022 and were primarily conducted in Asia (50.0%), Europe (20.0%), North America (20.0%) and Africa (10.0%). All of the participants were aged younger than 14 years. Nearly all participants (1,548 cases, 99.87%) had ASA status I and II, and only 2 cases (0.13%) were ASA III (32). The types of surgery of the 20 RCTs mainly include hernia repair, cleft lip and palate repair, tonsillectomy, strabismus surgery, otorhinolaryngology surgery, dental surgery, and so on. The main characteristics of the 20 studies are summarized in Table 1.

**Table 1:** Study characteristics.

Study	Country	Number of cases (Sevoflurane/ Propofol)	Age of cases (Years)	Type of operation	ASA class	Intervention measures		Outcome indexes
						Sevoflurane	Propofol	
König et al. (10)	USA	91/88	2–12	Dental surgery	I–II	2% sevoflurane	Propofol 250 µg/(kg·min)	EA, PONV, POP
Guard et al. (14)	Canada	25/25	2–8	Urinary surgery	I–II	2.5% sevoflurane	Propofol 5–10 mg/(kg·h)	PONV, TE, TEO, POP
Lopéz et al. (15)	Spain	60/60	1–12	Hernia repair	I–II	1.7% sevoflurane	Propofol 5 mg/(kg·h)	EA, POP
Picard et al. (16)	Switzerland	24/22	3–10	Tonsillectomy	I–II	2%–3% sevoflurane	Propofol 100–250 µg/(kg·min)	EA, PONV
Uezono et al. (17)	Japan	16/16	1–5	Eye examination	I–II	2%–4% sevoflurane	Propofol 100–400 µg/(kg·min)	PONV, TE, TEO
Kocak et al. (18)	Turkey	15/15	4–8	Otorhinolaryngology surgery	I–II	NA	NA	EA, TE, TEO
Kubo et al. (19)	Japan	28/23	4–8	Hernia repair	I–II	2%–3% sevoflurane	Propofol 100–400 µg/(kg·min)	EA
Cohen et al. (20)	USA	26/27	1–3	Outpatient surgery	I–II	1.5%–2.5% sevoflurane	Propofol 200 µg/(kg·min)	EA, PONV, TE, TEO, POP
Auerswald et al. (21)	German	27/27	1–5	Tonsillectomy	I–II	NA	NA	EA, PONV
Nakayama et al. (22)	Japan	89/87	2–11	Otorhinolaryngology surgery	I–II	1.5%–3% sevoflurane	Propofol 6–10 mg/(kg·h)	EA, PONV, TE
Deng et al. (23)	China	20/20	3–12	Cleft lip and palate repair	I–II	0.65–1.5 MAC sevoflurane	Propofol 4–10 mg/(kg·h)	EA, PONV, TE
Pieters et al. (24)	USA	19/19	3–7	Tonsillectomy	I–II	1.5%–4% sevoflurane	Propofol 150–300 µg/(kg·min)	EA, PONV, TE
Hasani et al. (25)	Kosovo	42/46	3–6	Hernia repair	I–II	1.5%–2% sevoflurane	Propofol 1 mg/(kg·h)	EA, PONV, TEO, POP
Cui et al. (26)	China	43/43	1–8	Hernia repair	I–II	2%–3% sevoflurane	Propofol 3 mg/(kg·h)	EA, TEO
Guo (27)	China	46/41	1–8	Hernia repair	I–II	2%–3% sevoflurane	Propofol 3 mg/(kg·h)	EA, PONV, TEO
Jiang (28)	China	26/26	1–8	Hernia repair	I–II	2%–5% sevoflurane	Propofol 6–8 mg/(kg·h)	EA, PONV, TEO
Omara et al. (29)	Egypt	40/40	0.5–1	Cleft lip and palate repair	I–II	2 MAC sevoflurane	Propofol 9 mg/(kg·h)	EA, TE, TEO
Dai (30)	China	35/35	1–8	Hernia repair	I–II	2%–3% sevoflurane	Propofol 3 mg/(kg·h)	PONV
Oriby et al. (31)	Egypt	42/42	3–11	Strabismus surgery	I–II	8% sevoflurane	Propofol 4 mg/(kg·h)	TEO, POP
Karam et al. (32)	Lebanon	69/65	0.5–7	Strabismus surgery, etc	I–III	8% sevoflurane	Propofol 5 mg/(kg·h)	EA, PONV

*ASA, American society of anesthesiologists; EA, emergence agitation; MAC, minimum alveolar concentration; NR, not record; PONV, postoperative nausea and vomiting; POP, postoperative pain; TE, time of extubation; TEO, time of eye opening.*

### Methodological Quality of Included RCTs

**Figure 2:**
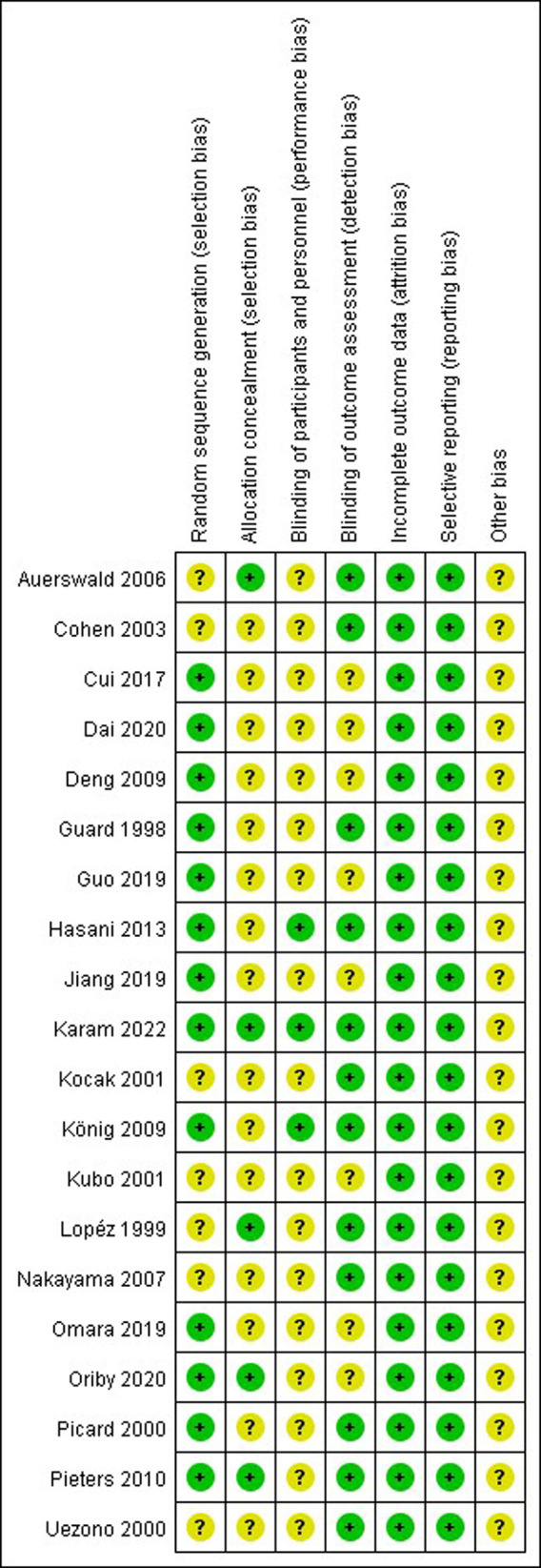
Methodological quality assessment of trials using the Cochrane risk of bias tool. Symbols show low risk of bias (+), or unclear risk of bias (?)

The methodologic quality item for the 20 included studies were described in Figure 2. Of the 20 studies, the methodological quality of most studies was limited. There were three randomized, double-blind clinical trials performed in children (10, 25, 32). Eleven studies used a random number table for randomization (10, 14, 16, 23–26, 28, 30–32), two studies used sealed opaque envelope (15, 24), one study used an online randomization program (29), and the other studies provide unclear information about the random sequence generation. In addition, most studies provide unclear information about the allocation concealment, blinding both participants and personnel, and blinding of outcome assessors. None of the 20 studies reported missing data.

### Assessment of the Primary Outcome

**Figure 3:**
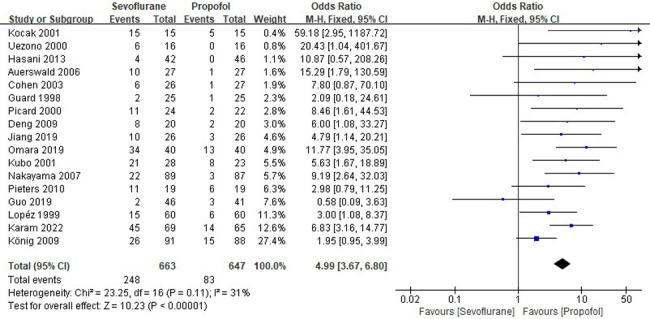
Pooled estimate of the incidence of emergence agitation (EA) between sevoflurane and propofol for general anesthesia in children. Odds ratio >1.0 indicates that the incidence of EA is lower in the propofol group than that in sevoflurane group. The subheading “Events” refers to the number of EA. “Total” refers to the total number of individuals. CI, confidence interval; df, degrees of freedom; M-H, Mantel-Haenszel method of calculation.

Seventeen RCTs tested the incidence of EA between sevoflurane groups and propofol groups after general anesthesia in children. As shown in Figure 3, a meta-analysis of the trials (*n* = 1,310) showed a significant increase of the incidence of emergence agitation for sevoflurane groups, compared to propofol groups (OR = 4.99, 95% CI, 3.67–6.80; *Z* test = 10.23, *P* < 0.00001). The *χ*^2^ test for homogeneity indicates that there are no statistically differences in results among the trials (*χ*^2^ = 23.25, df = 16; *P* = 0.11) with an I^2^ of 31% (I^2^ is typically considered low for <25%, modest for 25%–50%, and large for >50%), the fixed-effects model is used.

### Assessment of the Secondary Outcome

**Figure 4:**
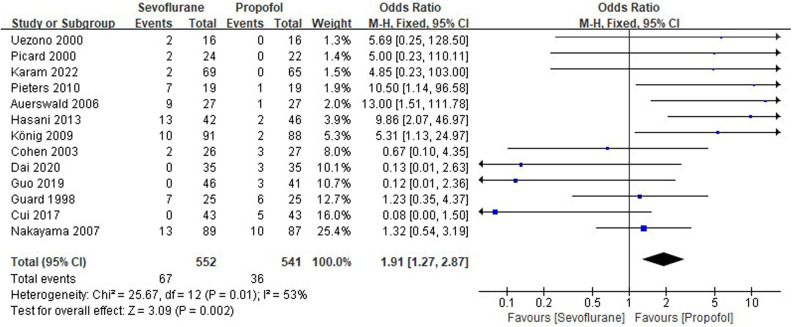
Pooled estimate of the incidence of postoperative nausea and vomiting (PONV) between sevoflurane and propofol groups for general anesthesia in children. Odds ratio >1.0 indicates that the incidence of PONV is lower in the propofol group than that in sevoflurane group. The subheading “Events” refers to the number of PONV. “Total” refers to the total number of individuals. CI, confidence interval; df, degrees of freedom; M-H, Mantel-Haenszel method of calculation.

Thirteen RCTs tested the incidence of PONV between sevoflurane groups and propofol groups after general anesthesia in children. As shown in Figure 4, a meta-analysis of the trials (*n* = 1,093) showed a significant increase of the incidence of postoperative vomiting for sevoflurane groups, compared to propofol groups (OR = 1.91, 95% CI, 1.27–2.87; *Z* test = 3.09, *P* = 0.002).

**Figure 5:**
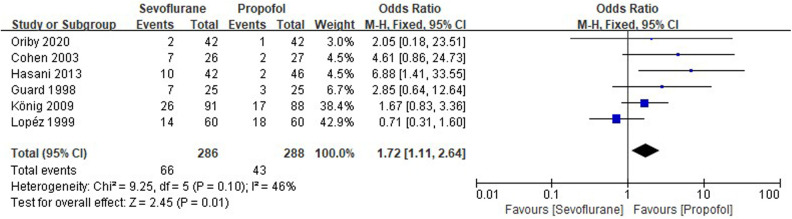
Pooled estimate of the incidence of postoperative pain (POP) between sevoflurane and propofol groups for general anesthesia in children. Odds ratio >1.0 indicates that the incidence of POP is lower in the propofol group than that in sevoflurane group. The subheading “Events” refers to the number of POP. “Total” refers to the total number of individuals. CI, confidence interval; df, degrees of freedom; M-H, Mantel-Haenszel method of calculation.

Six RCTs tested the incidence of POP between sevoflurane groups and propofol groups after general anesthesia in children. As shown in Figure 5, a meta-analysis of the trials (*n* = 574) showed a significant increase of the incidence of POP for sevoflurane groups, compared to propofol groups (OR = 1.72, 95% CI, 1.11–2.64; *Z* test = 2.45, *P* = 0.01).

**Figure 6:**
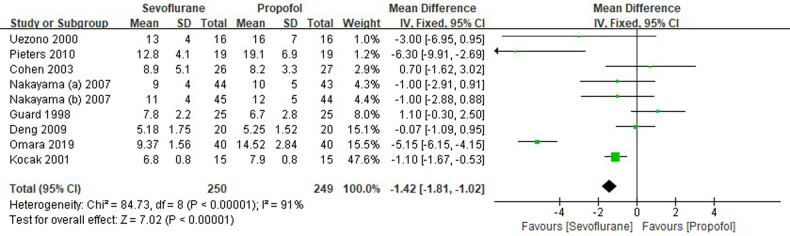
Pooled estimate of the extubation time (min) between sevoflurane and propofol groups for general anesthesia in children. Mean Difference < 0 indicates that the extubation time is shorter in the sevoflurane group than that in propofol group. The subheading “Total” refers to the total number of individuals. CI, confidence interval; df, degrees of freedom.

Eight RCTs tested the extubation time between sevoflurane groups and propofol groups after general anesthesia in children. As shown in Figure 6, a meta-analysis of the trials (*n* = 499) showed a significant decrease of the extubation time for sevoflurane groups, compared to propofol groups (MD = −1.42, 95% CI, −1.81– −1.02; *Z* test = 7.02, *P* < 0.00001).

**Figure 7:**
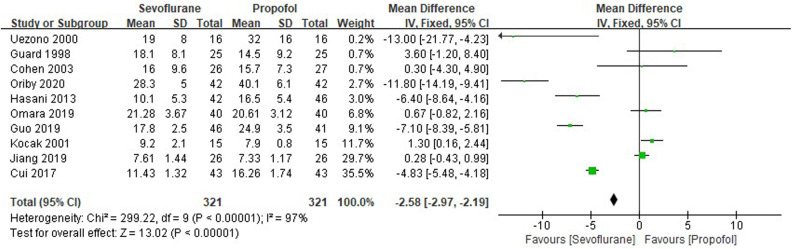
Pooled estimate of the time of postoperative eye-opening (min) between sevoflurane and propofol groups for general anesthesia in children. Mean Difference < 0 indicates that the time of postoperative eye-opening is shorter in the sevoflurane group than that in propofol group. The subheading “Total” refers to the total number of individuals. CI, confidence interval; df, degrees of freedom.

Ten RCTs tested the time of postoperative eye-opening between sevoflurane groups and propofol groups after general anesthesia in children. As shown in Figure 7, a meta-analysis of the trials (*n* = 642) showed a significant decrease of the time of postoperative eye-opening for sevoflurane groups, compared to propofol groups (MD = −2.58, 95% CI, −2.97– −2.19; *Z* test = 13.02, *P* < 0.00001).

### Publication Bias

**Figure 8:**
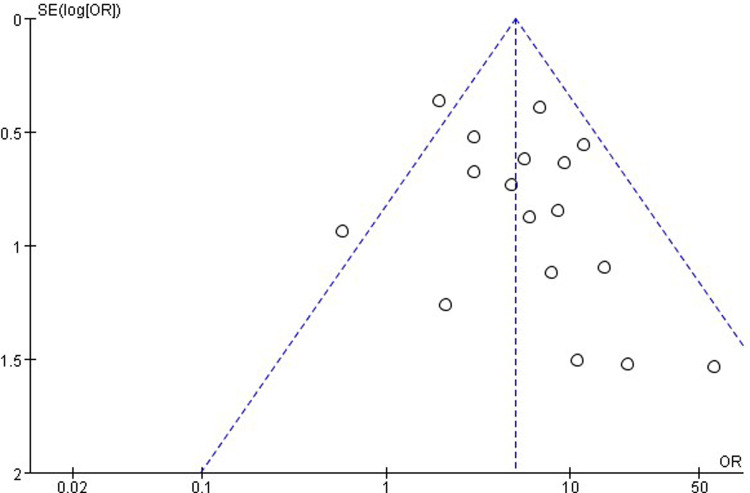
Funnel plots of randomized controlled trials.

The publication bias is important for interpreting the conclusions. As shown in Figure 8, the funnel plots of the incidence of EA showed that there was no publication bias.

## Discussion

In total, this study conducted a meta-analysis of RCTs to comprehensively evaluate the safety of propofol versus sevoflurane in children. According to predefined inclusion and exclusion criteria, 20 RCTs with 1,550 patients were included, but the quality assessment showed that most RCTs had low to moderate methodological quality. The results indicated that the use of propofol significantly decreased the risks of EA, PONV and POP in children, when compared with sevoflurane. However, the data also demonstrated that the paediatric patients who received sevoflurane tended to have shorter recovery times to eye opening and times to extubation.

EA is considered a postoperative behavioral disturbance, and is also a terminology describing nonpurposeful anxiety and restlessness, agitation, crying, and disorientation in the recovery stage of general anesthesia in pediatric patients (33, 34). EA is linked to increase the risk of self-harm and discomfort, and the costs for extra nursing care (35). Numerous studies have demonstrated that sevoflurane anesthesia is accompanied by a high risk of EA, and the incidence rate varies from 10% to 80% (36–38). Therefore, EA is a potential threat to children after sevoflurane anesthesia, and is also a general and difficult problem for anesthesiologists. A previous meta-analysis indicated that sevoflurane anesthesia in pediatric patients has a greater risk of EA than propofol anesthesia (39). Indeed, as shown in Figure 3, in the present meta-analysis, the occurrence rate of EA in patients with sevoflurane anesthesia is 37.40%, which is markedly higher (OR = 4.99, *P* < 0.00001) than in ones with propofol anesthesia (12.83%).

PONV has a high incidence in pediatric patients, especially for tonsillectomy and strabismus surgery (40–42). Recent studies had shown that PONV could lead to several adverse consequences, such as dehydration, electrolyte imbalance, and the wound dehiscence (43, 44). PONV can significantly delay discharge from hospital, lead to an unplanned hospital admission, and result in increased financial costs (45). As shown in Figure 4, in the present meta-analysis, the occurrence rate of PONV in patients with sevoflurane anesthesia is 12.14%, which is markedly higher (OR = 1.91, *P* = 0.002) than in ones with propofol anesthesia (6.65%).

POP is a common problem after pediatric surgery, and propofol has been shown to be associated with reduced POP compared with that associated with sevoflurane (24). As shown in Figure 5, the present meta-analysis suggests that patients receiving total intravenous anesthesia with propofol have been shown to experience less POP (OR = 1.72, *P* = 0.01), than receiving an inhalational anesthetic with sevoflurane. The result indicated that propofol produced an opioid-sparing effect and delayed first request of rescue analgesia.

In 2016, a previous meta-analysis of Peng et al. confirmed that sevoflurane anesthesia in pediatric patients had statistically greater risks of EA and PONV than propofol anesthesia (12), whereas there are no statistically differences in the incidences of POP (*P* = 0.16), times to eye opening (*P* = 0.28) and times to extubation (*P* = 0.16). The study of Peng et al. has not sufficiently presented meaningful differences between propofol and sevoflurane because the number of enrolled subjects is limited. Furthermore, it provided limited information regarding the search strategies, included and excluded criteria, and bias assessment, and did not address the overall quality of evidence. In the present meta-analysis, more RCTs (20 studies) and more participants (1,550 pediatric patients aged younger than 12 year) were included in this study. The results not only substantiated the previous findings for the incidences of EA and PONV, but also suggested that there are also statistically differences in results of the incidence of POP (*P* = 0.01), the times to eye opening (*P* < 0.00001) and the times to extubation (*P* < 0.00001). The results suggested that the children who received sevoflurane tended to wake earlier and have shorter recovery times.

Epileptiform discharges and cognitive impairment also reported occur in children during general anaesthesia (46, 47). The study of Koch et al. indicated that the occurrence rate of epileptiform discharges is 36% after propofol anesthesia in pediatric patients, which is 67% after sevoflurane anesthesia (46). The study of Fan et al. showed that the prolonged sevoflurane inhalation (≥3 h) significantly increased the risk of postoperative cognitive impairment (47). However, we were unable to investigate the risks of epileptiform discharges and cognitive impairment, because there were too few RCTs provided such detailed information.

In addition to the above advantages, there are also limitations for propofol anesthesia. First, propofol is an intravenous anesthetic, pain on injection is a major disadvantage. Many studies have been carried out to eliminate injection pain caused by propofol, but they are not successful yet (48). Secondly, it is necessary to establish a peripheral venous channel for propofol anesthesia. Some children who are not suitable for or refuse to receive intravenous infusion, sevoflurane is the first choice for anesthesia induction. In addition, the use of muscle relaxants in combination with propofol are present in all RCTs. Some studies have reported that sevoflurane may have similar and even better effects in children under the general anesthesia without muscle relaxant (49).

Finally, this study still has some limitations, which should be addressed. Frist, the included RCTs included various surgeries: hernia repair, cleft lip and palate repair, tonsillectomy, strabismus surgery, dental surgery, and so on. Different types of surgery required amount and duration of general anesthesia vary widely between procedures, which can lead to misinterpretation of the results. Secondly, 11 studies were published between 1998 and 2009, and 9 studies were published between 2010 and 2022, relatively few new studies have been initiated and reported in recent years. Third, 20 studies are included in this meta-analysis, only 3 (15.0%) randomized, double-blind clinical trials are found, 9 trials (45.0%) do not report the method of randomization, the great majority of the trials (55.0%) do not report the allocation concealment. The results can be substantiated to a limited degree, future studies are needed to address this issue with larger sample size and better methodological quality.

## Conclusions

In conclusion, the presently meta-analysis indicated that the children who received propofol anesthesia had the lower risks of EA, PONV and POP when compared with sevoflurane anesthesia. But the children who received sevoflurane anesthesia recovered faster than those received propofol anesthesia. Considering the limitations of the included studies, better methodological quality and large controlled trials are expected to further quantify the safety of propofol versus sevoflurane for general anesthesia in children.

## Data Availability

The original contributions presented in the study are included in the article/Supplementary Material, further inquiries can be directed to the corresponding author/s.
